# Draft Genome Sequence of a New Zealand Isolate of Mycoplasma ovipneumoniae

**DOI:** 10.1128/MRA.01375-19

**Published:** 2020-03-05

**Authors:** B. J. Bridgeman, S. K. Gupta, A. Murray, V. S. R. Dukkipati, E. Altermann, D. N. Wedlock

**Affiliations:** a School of Veterinary Science, Massey University, Palmerston North, New Zealand; b Animal Health, AgResearch, Hopkirk Research Institute, Grasslands Research Centre, Palmerston North, New Zealand; c School of Agriculture and Environment, Massey University, Palmerston North, New Zealand; d Riddet Institute, Massey University, Palmerston North, New Zealand; e AgResearch, Grasslands Research Centre, Palmerston North, New Zealand; University of Maryland School of Medicine

## Abstract

The genome of New Zealand Mycoplasma ovipneumoniae isolate 90 was sequenced and assembled using an Illumina MiSeq system and combining the built-in Geneious *de novo* and Velvet *de novo* assemblers. The 1,031,345-bp-long genome harbored 711 genes with a coding percentage of 86.6.

## ANNOUNCEMENT

Chronic nonprogressive pneumonia (CNP) is one of the most prevalent and difficult-to-treat diseases to infect sheep and young lambs in New Zealand, resulting in significant economic losses to the industry (1). Mycoplasma ovipneumoniae is the primary cause of CNP, reducing the host’s ability to prevent secondary infection by other pathogens. The draft genome presented here, designated isolate 90, is based on the sequence of a representative isolate from one of three dominant genotypes identified during a survey of farms in New Zealand which had a history of pneumonia.

Mycoplasma ovipneumoniae was isolated from pneumonic sheep according to a previously described method with slight modification (2, 3). Briefly, lungs from pneumonic sheep were washed with saline, and bronchoalveolar lavage fluid (BALF) samples were collected. BALF samples were inoculated into pleuropneumonia-like organism (PPLO) broth and incubated at 37°C until growth was evident as indicated by a phenol red pH indicator. Tenfold serial dilutions were prepared in PPLO broth from positive samples and incubated at 37°C. The highest dilution with a visible pH change was used for broth-to-plate cloning. A single drop of 25 μl of culture was layered onto PPLO agar plates. After incubation at 37°C for 72 h, colonies were observed under a stereo microscope. For plate-to-broth cloning, a single colony was picked by cutting out an agar plug using a sterile Pasteur pipette. This was inoculated into fresh PPLO broth and incubated at 37°C until a pH change was observed. The broth-to-plate and plate-to-broth cloning steps were repeated two more times to obtain pure cultures for each *M. ovipneumoniae* isolate.

Isolate 90 was cultured for sequencing in Frey’s medium (4) at 37°C until a phenol red pH indicator indicated growth of the mycoplasma. Genomic DNA was extracted using a ZR fungal/bacterial DNA miniprep commercial kit (Zymo Research Corp., Irvine, CA). The resulting genomic DNA was subsequently purified using a ZR genomic DNA clean and concentrator kit. DNA was sequenced on an Illumina MiSeq system (New Zealand Genomics Limited, Dunedin, New Zealand), producing 1,401,512 paired-end (PE) reads. The raw reads were trimmed against a UniVec database (Geneious R10.0.9) (5), and short-length reads (<25 nucleotides) were removed. After trimming, 1,214,927 PE reads (Geneious R10.0.9) (5) with an average length of 171 bases per read for a 200× coverage were used for genome assembly.

Default parameters were used for all software except where otherwise noted. The in-house Geneious R10.0.9 (5) and Velvet 1.2.10 (6) *de novo* assemblers were used to produce the initial assemblies, which had *N*_50_ values of 62 kbp and 38 kbp, respectively. The initial contigs were improved by remapping the sequence reads using the Geneious R10.0.9 map-to-reference function (5) and then using the in-house Geneious *de novo* assembler to combine the contigs into larger reads. Single nucleotide polymorphisms between the combined contigs were resolved by making a final map-to-reference run using the raw reads and taking the most common nucleotide. Combining the in-house Geneious (Geneious R10.0.9) (5) and Velvet 1.2.10 (6) reads produced 14 contigs.

The draft genome sequence was 1,031,345 bp long with a GC content of 29% and an *N*_50_ value of 85 kbp. The genome sequence was annotated using GAMOLA2 2.0.0.14 (7), and the gene model was built using Prodigal 2.6.3 (8). Gapped Blastp was carried out with the NCBI nonredundant amino acid database. Clusters of orthologous groups (COGs) were determined using the latest COG database (2014) (9). The CDD (v3.16) (10), Pfam (release 32) (11), and TIGRFAMs (release 15.0) (12, 13) databases were used to identify the respective domains. The annotation was visualized and analyzed using a modified version of Artemis 16 (7, 14, 15). In total, 711 genes with an average length of 1,257 bp and a coding percentage of 86.6 were identified.

### Data availability.

This whole-genome shotgun project has been deposited at DDBJ/ENA/GenBank under the accession number VZDP00000000. The version described in this paper is version VZDP01000000, and the BioProject number is PRJNA573593. The sequence reads have been deposited at the SRA under the accession number SRR10959050.
